# Modeling and Analysis of Spatial Inter-Symbol Interference for RGB Image Sensors Based on Visible Light Communication

**DOI:** 10.3390/s19224999

**Published:** 2019-11-16

**Authors:** Peng Liu, Peijun Zheng, Shiwei Yang, Ziyu Chen

**Affiliations:** Yangzhong Intelligent Electrical Institute, North China Electric Power University, Yangzhong 212200, China; zhengpeijun@ncepu.edu.cn (P.Z.); yangshiwei@ncepu.edu.cn (S.Y.); chenziyu@ncepu.edu.cn (Z.C.)

**Keywords:** optical wireless communication, image sensor communication, spatial inter-symbol interference, optimum detection threshold method

## Abstract

In this paper, RGB image sensors based on visible light communication system are designed and researched. In our study, the spatial inter-symbol interference is considered and the formation mechanism of the stray light, the influence of the spatial inter-symbol interference, and the influence of RGB image sensors are analyzed. The mathematics expression of the system signal-to-noise ratio (SNR) and bit error ratio (BER) is given. The simulation result indicates that there is a critical communication distance in the system. Once the communication distance exceeds the critical value, the system BER performance increases sharply. In addition, an adaptive threshold detection method is introduced and the performance is simulated. By means of estimating the spatial inter-symbol interference noise power, the optimal detection threshold can be obtained and the system BER performance increases significantly.

## 1. Introduction

With several advantages such as saving the radio frequency resource and being harmless to human beings, the visible light communication (VLC) technology has developed rapidly in the last decade. According to detection methods, the VLC system can be divided into a photodiode (PD) based on a VLC system and image sensors based on a VLC (IS-VLC) system [1]. Due to the widespread use of image sensors, the IS-VLC technology has a promising future in low-speed optical wireless communication, indoor positioning application, and intelligent transportation systems [2,3,4,5,6].

In order to improve the performance, a multi-input multi-output (MIMO) scheme is utilized in the IS-VLC system. Compared with the single-input single-output scheme, a MIMO-IS-VLC system can achieve not only a higher symbol transmission rate, but also a lower bit error rate [7]. In a MIMO-IS-VLC system, a frame of digital data stream drives the light-emitting diodes (LED) array where the on status of LEDs represents the data ‘1’ and off status corresponds to the data ‘0’. An image sensor used as a receiver captures a picture and sends it to be processed. By means of digital signal processing, every LED channel on the array is recognized and analyzed individually. After the demodulation, a frame of original digital data stream is obtained from the image [8].

However, there exists spatial inter-symbol interference (SISI) in a multi-input milti-output image sensors based on visible light communication (MIMO-IS-VLC) system that will degrade the system performance. In an ideal imaging system, the light reaching the imaging plane is the target light of imaging objects. However, there will be non-target stray light in a practical imaging system, leading to the interference among different LED channels.

The stray light is defined as the irregular incident light of the imaging object. It is the major factor contributing to the SISI. The LED of an on status has similar light intensity contour distribution that can be equivalent to concentric circles. However, in the region where LEDs of on status are relatively concentrated, the outside surfaces of every concentric circle will connect with each other and form a larger area. It reveals the fact that, in the MIMO-IS-VLC system, different LED channels will influence each other and produce the SISI. From the point of view of an optical wireless communication system, the spread distribution of LEDs’ light intensity on the imaging plane gives rise to the SISI and degrades the system performance. The paper [9] studies the impacts of SISI on the visual MIMO system’s capacity from the perspective of the imaging process. However, the generation mechanism of SISI is not discussed, and the further analyses such as the estimation of the SISI noise intensity is not provided.

Many methods have been proposed to enhance the speed and accuracy of light source detection of optical wireless communication systems. The authors of [10] design a Complementary Metal-Oxide-Semiconductor (CMOS) image sensor communication chip that realized the high-speed and long-distance spatial optical communication over 10 Mb/s and 50 m. The key technique of the presented chip is a new pixel structure using depleted diode, pulse equalizing, and binary flag image readout to find the exact area of light source. In [11], an optical wireless communication system based on an LED transmitter and a camera receiver along with an optical communication image sensor is proposed. To obtain higher transmission rates, the optical communication sensor employs a specialized communication pixel that is capable of responding promptly to optical intensity variations. The paper [12] introduces an optical wireless communication technology based optical vehicle to vehicle communication systems linked by an LED transmitter and a camera receiver. An optical communication image sensor is employed in the camera receiver, which has a non-conventional pixel specialized for high-speed optical signal reception. For the image frames received by image sensors, the pixel rows are activated from top to bottom. If the modulation speed of the LED is faster than the frame rate of the CMOS image sensor, the bright and dark stripes will be respectively clipped in the image frame representing the LED on and off. The advantage of this information reception method is that the receiver can accurately locate the transmitter according to the transmitter’s frequent frame rate changes. In [13], a modified adaptive scheme for the demodulation of the CMOS image sensor rolling shutter pattern is introduced. The results show that this scheme can provide a much lower bit error rate, with similar process latencies with other schemes. In [14], a modulation scheme in the time domain based on On-Off-Keying is proposed and various compatible supports for different types of image sensors are given. The proposed frame structures and decoding systems for the oversampling prototype and undersampling prototype can effectively mitigate the frame rate variation. In [15], a new distance estimation technique employing photography and image sensor communications is presented and analyzed. The proposed system includes a non-flickering visible light system transmitter, an optical camera communication rolling shutter receiver, and employs a photogrammetry mechanism for distance estimation.

The purpose of this paper is to reduce the effects of stray light, caused by diffraction, refraction, and reflection of incident light on the system, thereby reducing spatial inter-symbol interference. By estimating the SISI noise power, an optimal detection threshold is obtained, resulting in a significant increase in system bit error rate (BER) performance.

For the purpose of modeling and analyzing the SISI in a MIMO-IS-VLC system, a more detailed system description is given in Section 2. Then, the generation mechanism of the stray light and the mathematical description are discussed. In Section 3, the mathematical expression of the SNR and the BER of MIMO-IS-VLC system with SISI is deduced, and the corresponding simulation result is demonstrated and analyzed. The conclusions are obtained in Section 4.

## 2. The System Model

Currently, most receiver systems use color cameras, which makes it necessary to consider the problem of color image changes caused by a three-color receiving system of RGB images. In Section 2.1, a brief description of a MIMO-IS-VLC system is given. In Section 2.2, we analyze the effect of stray light on a MIMO-IS-VLC system. In Section 2.3, the channel model of a MIMO-IS-VLC system is introduced. In Section 2.4, the influence of color sensors is discussed.

### 2.1. System Description

**Figure 1:**
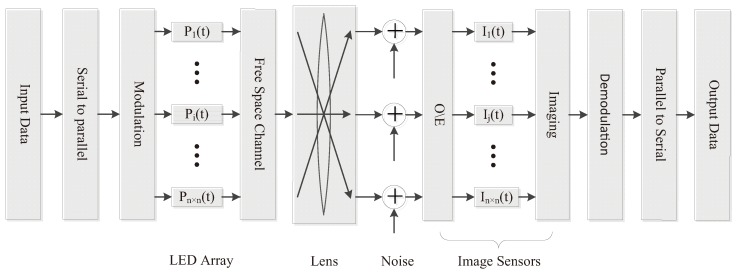
The block diagram of a multi-input milti-output image sensors based on visible light communication system.

The block diagram of MIMO-IS-VLC system is shown in Figure 1. Firstly, a frame of serial input data is converted to the parallel data and drives a n×n LED array by On-Off key (OOK) modulation scheme. According to the given protocol, each digital signal channel controls a specific LED on the array and maintains independence from each other.

Assuming that $P_{LED}$ is the rated optical power of LEDs and $s_{i}\left(t\right)$ is the input digital signal of the *i* channel, there is:

(1) $$P_{i}\left(t\right)=\left\{\begin{array}{cc} P_{LED}, & s_{i}\left(t\right)=1,\\ 0, & s_{i}\left(t\right)=0, \end{array}\right.$$

where 1≤i≤n×n, $P_{i}\left(t\right)$ is the *i* channel’s LED transmitting optical power.

Secondly, a high speed charge-coupled device (CCD) or CMOS device with a lens captures the picture of the LED array and transfers it to be processed. The image processing algorithm segments the picture and every single LED channel is analyzed individually. Then, the LED optical signal is demodulated to digital signal and an n×n data matrix is generated.

Finally, after the parallel-to-serial conversion, a frame of the transmitted serial data is obtained.

### 2.2. Distribution Model of the Stray Light

The spatial inter-symbol interference of the MIMO-IS-VLC system is mainly caused by the stray light. The stray light can be considered as an additive background noise component to the system. There are two main sources of the stray light: one is the external light such as the sunlight, the indoor illumination light, and the atmospheric diffusion light. It is considered as a constant background light, and the light intensity value can be expressed as *K*. The other is the irregular incident light of the imaging object. In the ideal condition, the incident light of the imaging object follows a certain angle. However, a part of imaging light reaches the image sensor at an uncertain angle and leads to the disorder of imaging process. In particular, there are three reasons accounting for the irregular incidence:

- The diffraction caused by the grating edge and the grating slit takes place.
- The refraction takes place because the camera lens is contaminated, and the surface of the optical components such as gratings, prisms, lenses, filters, and others is not flat.
- The reflection takes place on the inner wall of the tube.

**Figure 2:**
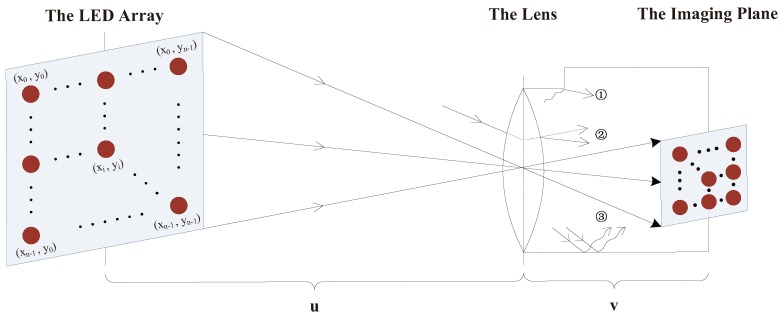
The optical path of the imaging light in the MIMO-IS-VLC system. (**a**) diffraction light at the edge of the grating; (**b**) refraction light from the lens; (**c**) reflection light on the inner wall of the lens’ tube.

For the image sensor, the incident angle of the stray light is stochastic, but its spatial distribution is regular. According to [16], the spatial distribution of stray light can be described as the Kirk model. As is shown in Figure 2, the rectangular coordinate system based on one of four corners’ LEDs is established, and $\left(x_{0},y_{0}\right)$ is set as the original point. Then, the point spread function (PSF) of stray light is written as

(2) $$s\left(r_{i}\right)=\frac{1}{\sigma \sqrt{2\pi }}exp\left(-\frac{r_{i}^{2}}{2\sigma^{2}}\right),$$

where $r_{i}=\sqrt{x_{i}^{2}+y_{i}^{2}}$ is the distance from the point $\left(x_{i},y_{i}\right)$ to the original point, σ is the intensity coefficient of the stray light distribution. Assuming $P(x_{0},y_{0})$ is the light intensity of the original point. Thus, to a certain area based on the center coordinate of $\left(x_{i},y_{i}\right)$, the stray light intensity is written as

(3) $$S\left(x_{i},y_{i}\right)=K+\;\int \int \;P\left(x_{0},y_{0}\right)\cdot s\left(x-x_{i},y-y_{i}\right)\;dx\;dy,$$

where *K* is a constant background light intensity, *P*($x_{0}$, $y_{0}$) is the rated optical power of the origin, and *s*($x-x_{i}$, $y-y_{i}$) is the PSF of stray light.

When calculating the influence of stray light in the imaging system, the exposure time $t_{e}$ should be considered. The total exposure value $P_{v}$ is the time integral of the instantaneous light intensity *P* and is expressed as $P_{v}=\int P\;dt$. Because the stray light distribution function is not a time correlation function, the total exposure energy can be simply written as $P_{v}=P\cdot t_{e}$.

### 2.3. Channel Model

Assuming $S\left(t\right)=(s_{1}\left(t\right),\ldots ,s_{i}\left(t\right),\ldots ,s_{n\times n}\left(t\right)),\left(1\leq i\leq n\times n\right)$ is a frame of the original serial digital data and based on Equation (1), the output optical signal of the LED array is $P={\left(P_{1}\left(t\right),\ldots ,P_{i}\left(t\right),\ldots ,P_{n\times n}\left(t\right)\right)}^{T}$, the received current signal of the image sensor $I={\left(I_{1}\left(t\right),\ldots ,I_{i}\left(t\right),\ldots ,I_{n\times n}\left(t\right)\right)}^{T}$, which can be written as

(4) I=ξH×P+N,

where ξ is the photoelectric conversion coefficient of the image sensor, H is the channel gain matrix of n×n order, and $N={\left(\delta_{1},\ldots ,\delta_{n\times n}\right)}^{T}$ is the system noise matrix of n×n order.

Considering the channel as a line of sight (LOS) link, the MIMO channel gain matrix is composed of the link gain G and the SISI gain S, which means H=G+S. Both of them are n×n matrices. In addition, the link gain matrix G is a diagonal matrix, and the SISI gain matrix’s diagonal elements are zero.

Figure 2 shows a geometric graph of the MIMO-IS-VLC system. The focal length of lens is *f*. *u* is the object distance and *v* is the image distance. *d* is the spacing distance of two adjacent LEDs in the array. On the basis of the imaging system, there is u>2f and f<v<2f so that a real and miniature image of the object is acquired on the imaging plane.

The LED light radiation follows the Lambertian model. Thus, the diagonal elements of G can be written as [17]:

(5) $$g_{ii}=\left\{\begin{array}{cc} \frac{A}{u^{2}}\;\;R\left(\phi \right)\cdot cos\left(\psi_{i}\right), & 0\leq \psi_{i}\leq \frac{\psi_{c}}{2},\\ 0, & \psi_{i}>\frac{\psi_{c}}{2}, \end{array}\right.$$

where *A* is the aperture area, $R\left(\phi \right)=\left[\left(m+1\right)/2\pi \right]cos^{m}\phi$, and ϕ is the LED irradiance angle. $\psi_{i}$ is the incident angle of *i*th LED. $m=-ln2/ln(cos\psi_{1/2})$, *m* is the order of the Lambertian radiation. $\psi_{1/2}$ is the emission angle at half power of the LED. $\psi_{c}$ is the angle of view of the lens.

Establishing the rectangular coordinate system based on one of the LEDs in the array, the distance of *i*th and *j*th LED can be expressed as $r_{ij}=d\cdot \sqrt{{\left|x_{i}-x_{j}\right|}^{2}+{\left|y_{i}-y_{j}\right|}^{2}}$. Under the condition that the object distance *u* is much larger than the image distance *v*, there is v≈f. Hence, the spacing distance of two adjacent LEDs in the imaging plane can be expressed as

(6) $${r_{ij}}^{\prime }=\frac{f\cdot r_{ij}}{u},$$

where $r_{i}j$ is the distance of *i*th and *j*th LED, *f* is the focal length of lens, *u* is the object distance. From Equation (3), the off-diagonal elements of S can be written as

(7) $$S_{ij}=\;\int \int \;P\left(x_{j},y_{j}\right)\cdot s\left({r_{ij}}^{\prime }\right)\;dx\;dy$$

where $P\left(x_{j},y_{j}\right)=g_{jj}\cdot P_{j}\left(t\right)$.

The additive noise in the MIMO-IS VLC system includes the constant stray light noise, the thermal noise, the shot noise, and the readout noise. Since the noise of the latter three is much less than the constant stray light noise, the additive noise in system of the *i* channel is expressed as $\delta_{i}=K$. As a result, the received current signal of *i*th channel is obtained by

(8) $$I_{i}\left(t\right)=\xi t_{e}\left\{g_{ii}\cdot P_{i}\left(t\right)+\sum_{j=1,j\neq i}^{n\times n}S_{ij}\cdot P_{j}\left(t\right)+K\right\}.$$

### 2.4. Influence of Color Sensor

Since LEDs have a faster switching rate, higher robustness, and higher reliability than lamps, LED as a light source can replace incandescent light sources [18]. Limiting the readout of the images to region of interest allows an increase in the frame rate of the images. In normal cameras, different resolutions correspond to different frame rates. If you want to increase the frame rate, you need to consider whether to narrow the field of view (FOV) or not reduce the resolution. Two common ways to reduce resolution are skipping and binning. In addition, since the limitation in readout data rate of image sensor and video output format are fixed, CMOS sensors must be transferred to the corresponding video output specification by corresponding methods. Different video formats are scanned and sub-sampled differently. Table 1 shows common video format. Skipping reduces the resolution by selecting the pixels in the field of vision and extracting the designated pixels. In skipping mode, the pixels of all rows and columns are not sampled, so that non-original resolution images (images with reduced resolution) can be obtained. Row and column data are read in pairs. Next, we will only study the image color distortion caused by skipping with equal spacing skipping (SVGA) and unequal spacing skipping (CIF).

**Figure 3:**
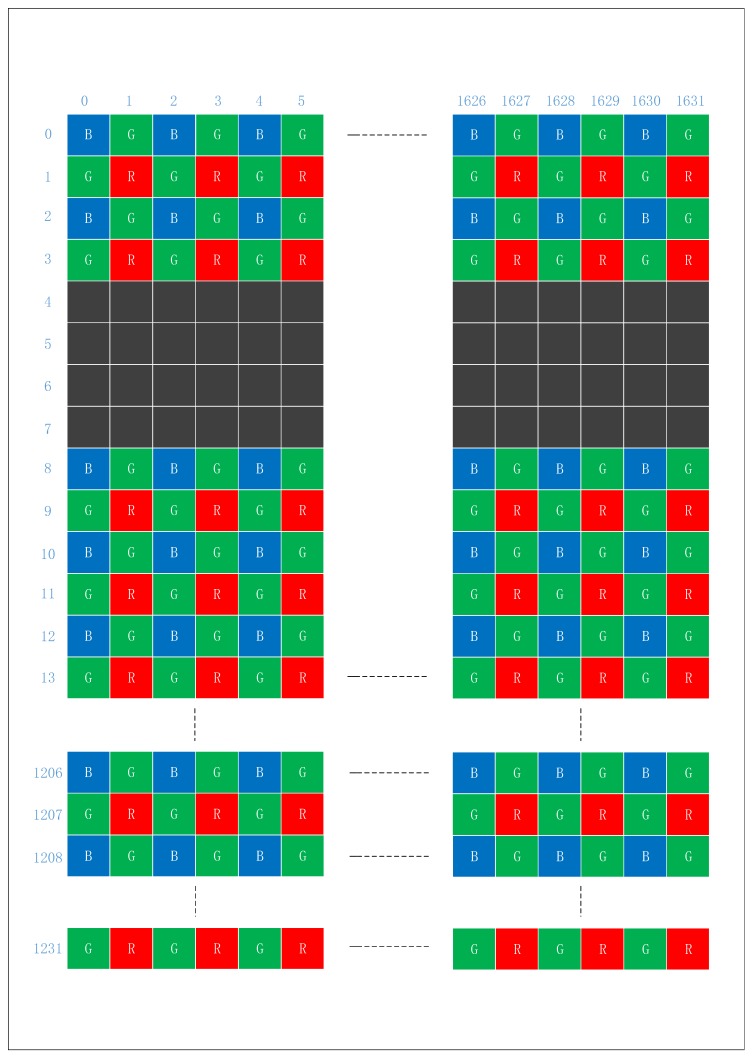
The structure diagram of the RGB sensor.

Figure 3 is the structure diagram of the RGB sensor. Each basic unit is made up of four pixel units, including two Gs, one B and one R. A picture is obtained by arranging the positions of four pixel units.

**Figure 4:**
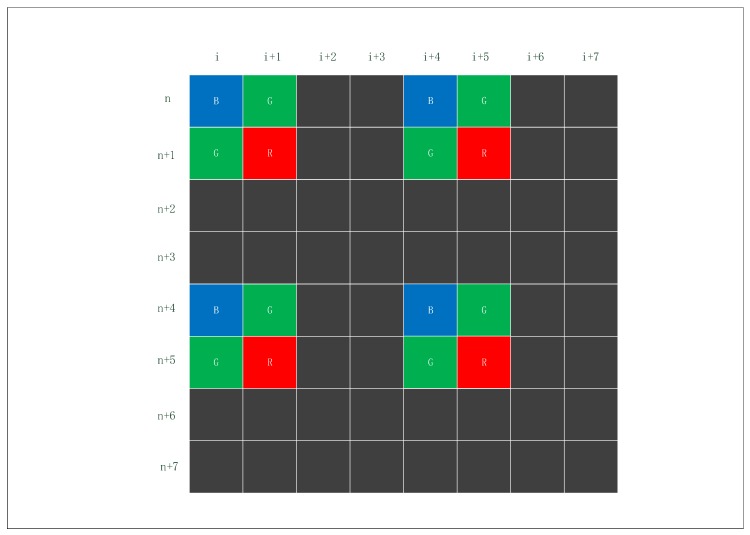
The schematic diagram of 2:2 skipping.

In 2:2 skipping, both horizontal and vertical pixels will be sub-sampled by taking two pixels every four pixels, as shown in Figure 4. With this option of the SVGA, this process reduces the image resolutions by half.

**Figure 5:**
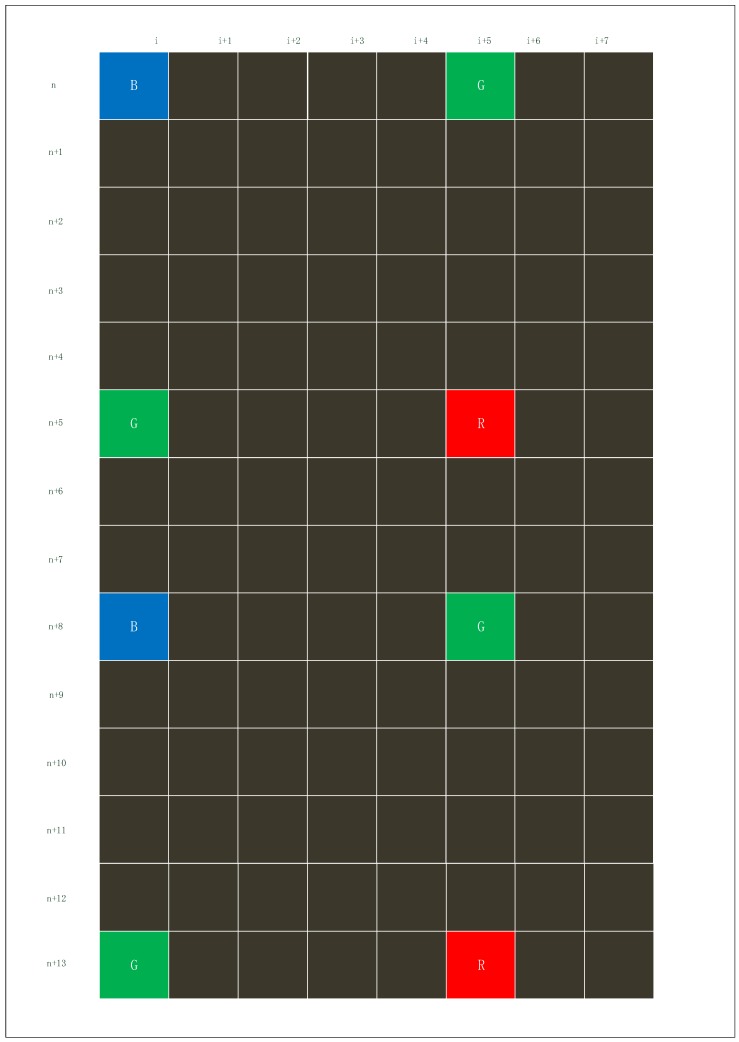
The schematic diagram of 4:4 skipping.

In 4:4 skipping, the edge of the pixel in four units will be sub-sampled, and four adjacent pixels are binned into one larger pixel and read out, as shown in Figure 5. This process reduces the image resolutions by 3/4. It takes less readout time to capture a color image than SVGA does. Figure 4 shows an example of the 2:2 skipping. (n,i),(n,i+1),(n+1,i),(n+1,i+1) are taken as the first effective unit. Analogously, (n+4,i+4),(n+4,i+5),(n+5,i+4),(n+5,i+5) are the second effective unit. The image is not deformed because the way of sub-sampling is proportional.

Figure 5 shows an example of the 4:4 skipping. (*n*, *i*), (*n*, *i* + 5), (*n* + 5, *i*), (*n* + 5, *i* + 5) are taken as the first, second, third, and fourth pixel point of the new unit. It can be concluded from the figure that processed effective pixels are not evenly spaced, so the images are distorted.

**Figure 6:**
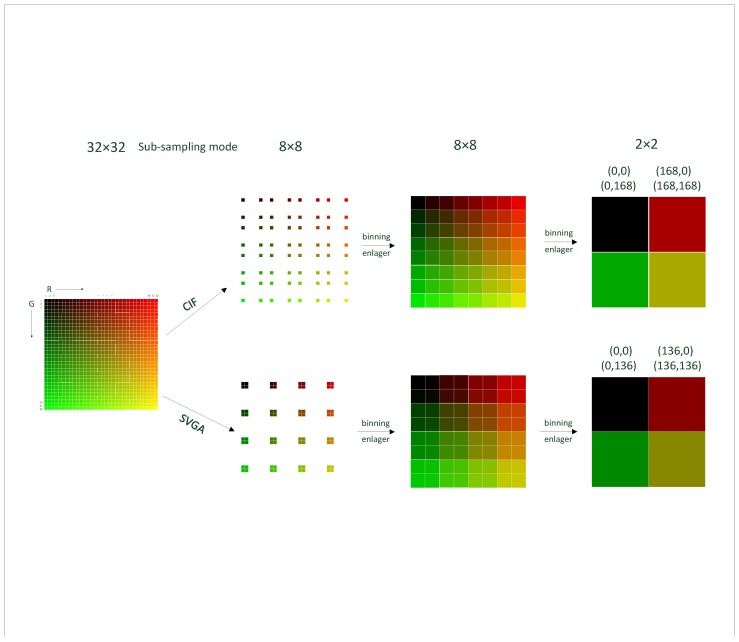
Color distortion of 4:4 skipping and 2:2 skipping.

The sampling process and the color contrast of the image after applying the two skipping modes mentioned above comes next. In the 32×32 original image, the value of channel R horizontally increases by 8, and the value of channel G vertically increases by 8. Figure 6 shows that the colors in the final 2×2 image are different due to the employment of two skipping ways. The first half of the image is sampled by unequal spacing skipping method (as shown in Figure 4), and the second half is sampled by equal spacing skipping method (as shown in Figure 5). Firstly, by comparing two different sampling methods, it can be found that CIF needs two sampling times in the process of obtaining 2×2 final image from the 32×32 original image, and SVGA needs four sampling times. CIF has fewer sampling times, so it takes less time to read the color image. At the same time, by comparing the two methods because CIF sampling is skipping with unequal spacing, the color distortion and image distortion caused by CIF sampling are larger, and each method has its own advantages and disadvantages.

As mentioned earlier, color distortion is caused by the sub-sampling in SVGA mode and CIF mode, and image deformation also caused by the sub-sampling in CIF mode, as shown in Figure 3. Meanwhile, the pixel skipping technology can produce obvious color distortion when compensating image details.

To summarize, there are some questions yet to be researched:

- When the color sensor communicates, a processed skipping image will have a greater impact on the color cast of the image.
- The effect of RGB three-channel resolution on information transfer.
- The extent to which color distortion and image deformation are acceptable in order to alleviate the crosstalk between both pixels.

## 3. The Simulation and Analysis

### 3.1. The SNR and BER

Assuming that the optical active area of the LED is circular and the radius is Ra, the projective radius of the LED optical active area on the imaging plane can be obtained from Equation (6) as ${Ra}^{\prime }=\frac{f\cdot Ra}{u}$. Combined with the Equations (2), (7) and (8), the received SISI noise of the system $\left(0\leq \psi_{i}\leq \psi_{c}\right)$ is obtained as

(9) $$I_{SISIN}=\xi t_{e}\sum_{i=1}^{n\times n}\sum_{\begin{array}{c} j=1\\ j\neq i \end{array}}^{n\times n}\int_{0}^{2\pi }d\theta \int_{0}^{{Ra}^{\prime }}\frac{g_{jj}\cdot P_{j}\left(t\right)}{\sigma \sqrt{2\pi }}exp\left(-\frac{{r_{i}^{\prime }}^{2}}{2\sigma^{2}}\right)\;dr.$$

The SISI noise component is an additive noise to the system. Equation (9) indicates that the SISI noise component mainly depends on the spacing distance between two adjacent LEDs in the imaging plane ${r_{ij}}^{\prime }$. In other words, three major factors, including the communication distance *u*, the lens’ focal length *f*, and the spacing distance of two adjacent LEDs $r_{ij}$ in the LED array, work together to influence the system BER performance based on Equation (6). As a result, with given communication distance and lens, the probability of received SISI noise intensity follows the function of ${r_{ij}}^{\prime }$.

The average expectation value of ${r_{ij}}^{\prime }$ can be calculated by permutation and combination theory. Under the circumstance that $P\left[s\left(t\right)=0\right]=P\left[s\left(t\right)=1\right]=\frac{1}{2}$, the average expectation intensity of SISI noise can be estimated. For an n×n channels MIMO-IS-VLC system, ${r_{ij}}^{\prime }$ is not an integer multiplier of the LED spacing distance *d*. Based on the permutation and combination theory, the average expectation value of ${r_{ij}}^{\prime }$ is calculated as

(10) $$r^{\prime }\left(n,q\right)=\left\{\begin{array}{cc} \frac{C_{2}^{1}C_{n}^{1}C_{n-q}^{1}}{C_{n\times n}^{2}}\cdot \frac{qdf}{u}, & Ra=qd,\\ \frac{C_{2}^{1}C_{2}^{1}C_{n-q}^{1}\left(\sum_{p=1}^{t}C_{n-p}^{1}\right)}{C_{n\times n}^{2}}\cdot \frac{\sqrt{q^{2}+p^{2}}df}{u}, & Ra\neq qd, \end{array}\right.$$

where q,p∈N, and 0<q≤n, Ra is the radius. Combined with Equation (9), the total expectation received SISI optical intensity is estimated as

(11) $$\begin{array}{cc} I_{SISIN} & =G\sum_{q=1}^{n-1}\sum_{p=1}^{q}\left[2n\left(n-q\right)exp\left(-\frac{{\left(qdf\right)}^{2}}{2\sigma^{2}u^{2}}\right)\right.\\ & \left.+4\left(n-q\right)\left(n-p\right)exp\left(-\frac{\left(q^{2}+p^{2}\right)d^{2}f^{2}}{2\sigma^{2}u^{2}}\right)\right], \end{array}$$

where $G=\frac{\xi g_{jj}\cdot P_{LED}\cdot t_{e}\cdot \pi Ra^{\prime 2}}{\sigma \sqrt{2\pi }}$. Equation (11) is a summation of the total expectation $I_{SISIN}\left[r^{\prime }\left(n,q\right)\right]$. Therefore, the signal-to-noise ratio (SNR) of the receiving terminal can be calculated as $R_{SN}=\sum_{i=1}^{n\times n}{\left[GP_{i}\left(t\right)\right]}^{2}/\left[{I_{SISIN}}^{2}+{\left(\xi K\right)}^{2}\right]$.

The MIMO-IS-VLC system can be considered as a unipolar OOK modulation system. From Ref. [19], it is known that the ISC system’s noise distribution obeys Gaussian distribution and signal dependence. Assuming that the constant stray light component *K* is equal to the background noise light and the one-dimensional probability density of the signal sampling value is a Gaussian random variable, the one-dimensional probability density when the transmitting signal is ‘1’ can be expressed as

(12) $$f_{1}\left(x\right)=\frac{1}{\sigma_{n}\sqrt{2\pi }}exp\left\{-\frac{{\left(x-P_{LED}\right)}^{2}}{2\sigma_{n}^{2}}\right\}.$$

When transmitting signal ‘0’, the one-dimensional probability density can be expressed as

(13) $$f_{0}\left(x\right)=\frac{1}{\sigma_{n}\sqrt{2\pi }}exp\left\{-\frac{{\left(x-\sigma_{b}-\sigma_{SISI}\right)}^{2}}{2\sigma_{n}^{2}}\right\},$$

where $\sigma_{SISI}$ and $\sigma_{b}$ are the variance of SISI noise and background noise’s sampling value correspondingly. There is ${\sigma_{SISI}}^{2}={I_{SISI}}^{2}$, ${\sigma_{b}}^{2}={\left(\xi K\right)}^{2}$ and $\sigma_{n}=\sqrt{{\sigma_{SISI}}^{2}+{\sigma_{b}}^{2}}$. Thus, with a given detection threshold Th $\left(0<Th<P_{LED}\right)$, the BER of the system can be obtained as

(14) $$P_{e}=\frac{1}{2}\left[erfc\left(T-\frac{1}{\sqrt{2}}\right)+erf\left(T-\sqrt{R_{SN}}\right)\right],$$

where *T* is normalized detection threshold and $T=\frac{Th}{\sigma_{n}\sqrt{2}}$. The optimal detection threshold can be defined by finding the extreme value of $\frac{\partial P_{e}}{\partial T}=0$. As a result, the optimal detection threshold is obtained as

(15) $$Th=\frac{GP_{LED}+I_{SISI}+\xi K}{2}.$$

### 3.2. Experimental Setup and the Simulation Results

**Figure 7:**
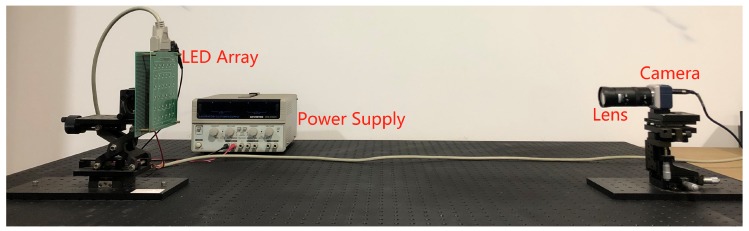
The experimental device for the MIMO-IS-VLC system.

In this section, the experimental setup for the MIMO-IS-VLC system is demonstrated. As shown in Figure 7, the experimental VLC setup is composed of LED array, power supply, lens, and camera. In addition, Table 2 presents some parameters of the experimental device.

**Figure 8:**
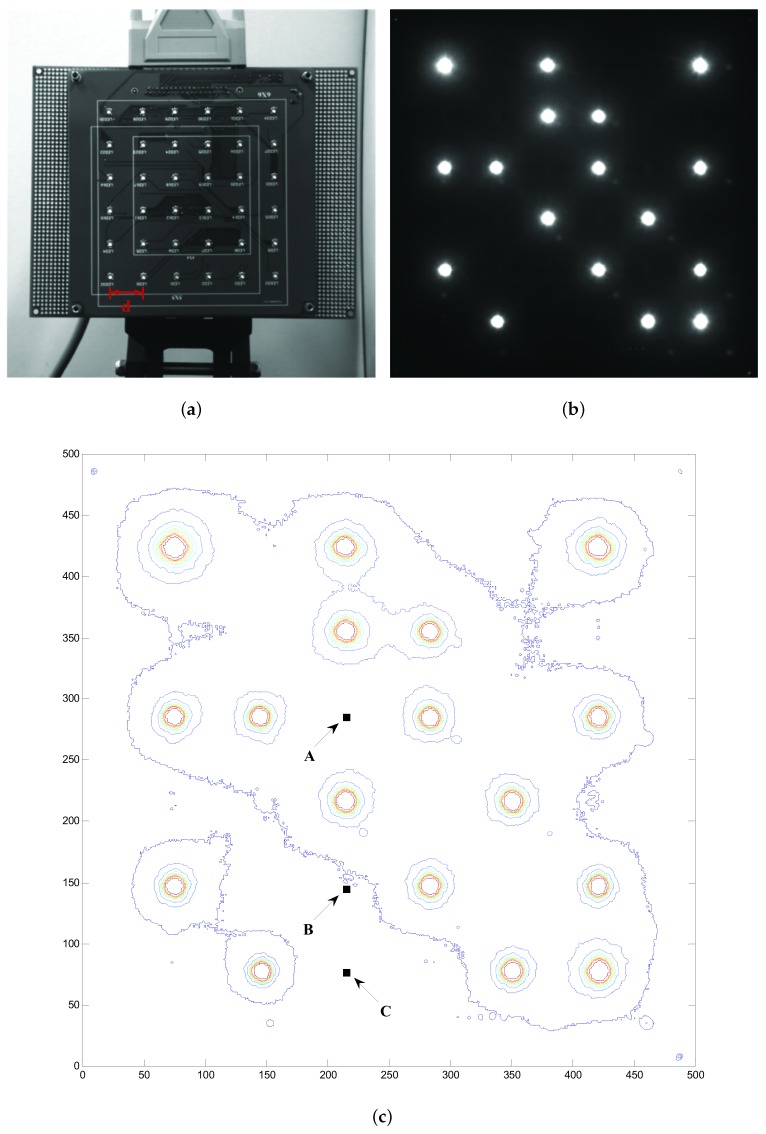
Demonstration of the spatial inter-symbol interference. (**a**) the designed experiment board with 6×6 LED array and the LED spacing distance *d*; (**b**) imaging result of the LED array; (**c**) light intensity contour plot of (**b**).

The distance between LED illumination is d (1.8 cm), as shown in Figure 8a. Figure 8b is the effect diagram. As is shown in Figure 8c, the LED of on status has similar light intensity contour distribution which can be equivalent to concentric circles. A, B, and C in Figure 8c are the positions of the LEDs of off status. According to the light intensity contour, the light intensity value of LED on position A is higher than that of the other two positions and the light intensity value of LED on C is the lowest. B is on the boundary of light intensity contour among A and C. The interference light intensity from other LED channels makes it easier for an LED to be recognized as an on status LED when applying a constant judgment threshold to demodulate the signal. From the point of view of an optical wireless communication system, the spread distribution of LEDs’ light intensity on the imaging plane gives rise to the SISI and degrades the system performance.

**Figure 9:**
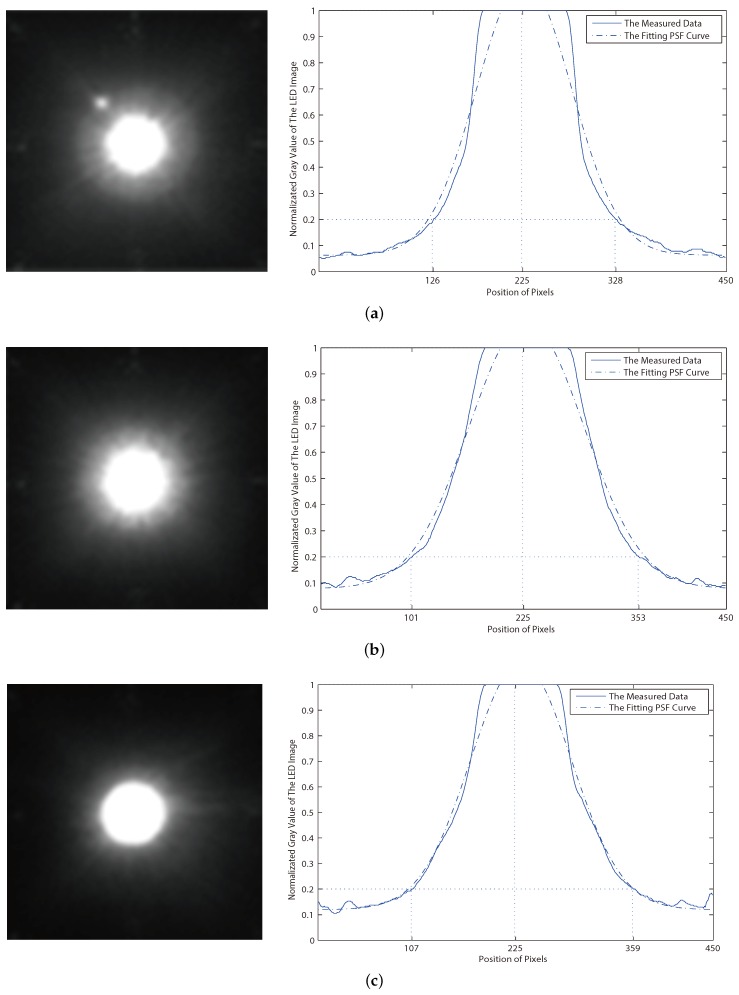
The imaging results of three different lenses under the same photography conditions.

In the experiment, we get imaging results of three different lenses and the fitting curve of the stray light intensity distribution as shown in Figure 9. The LED and the lens of image sensor are kept in parallel as much as possible to eliminate aberrations. The pixels on an image’s horizontal line are analyzed. For the purpose of increasing accuracy, the horizontal line from different angles and averaging is selected. As is shown in Figure 9, the horizontal coordinate is the pixel’s position from 0 to 450, and the vertical coordinate is the normalized gray value. An industrial CMOS camera is utilized in the experiment. The 3W white LED is at a rated illumination status. The three lenses come from different manufacturers with the same parameters. The diameter of the three lenses is 40 mm. In the experiment, the focal length of lenses is set as 20 mm and the aperture is F1.6. The exposure time $t_{e}$ is 10 ms, and the distance from the LED to the image sensor is 1 m. The pixel size of the original image is 450×450.

It can be seen that, under the same photograph condition, three lenses show differences in the imaging performance. To evaluate the concentrating degree of the light intensity distribution, set 0.2 as a threshold value of the normalized gray value and define the range in which the gray values of the pixels exceed 0.2. The narrower the range that the pixels’ distribution is, the more concentrated the light intensity distribution will be. In Figure 9a, the range of horizontal coordinate is (126, 328) and the normalized gray-value is higher than 0.2. It means that 80% of the pixels formed by the incident light are concentrated on around 45% pixels of the whole image. However, the range is (101, 353) and the proportion is around 56% in Figure 9b, while, in Figure 9c, the range is (107, 359) and the proportion is also 56%. It demonstrates that imaging light of the LED is more concentrated in Figure 9a than the other two.

With the purpose of obtaining the parameter σ in Equation (2), the least square method is used to get the fitting curve of the light intensity distribution. As a result, the obtained σ of the curve from Figure 9a is 86.2, while it is 95.1 of Figure 9b and 90.2 of Figure 9c (The adjusted *R*-square value of the fitting curve in three images is 0.984, 0.980 and 0.985 correspondingly). A smaller σ value of the Equation (2) leads to a narrower distribution range of the stray light, thus it is proved that the imaging pixel of the LED is more concentrated in Figure 9a. It also comes to the conclusion that, under the same condition, the lens with a smaller σ value of the stray light PSF has higher imaging performance because the imaging light is more concentrated, and the imaging result suffers less from the stray light.

**Figure 10:**
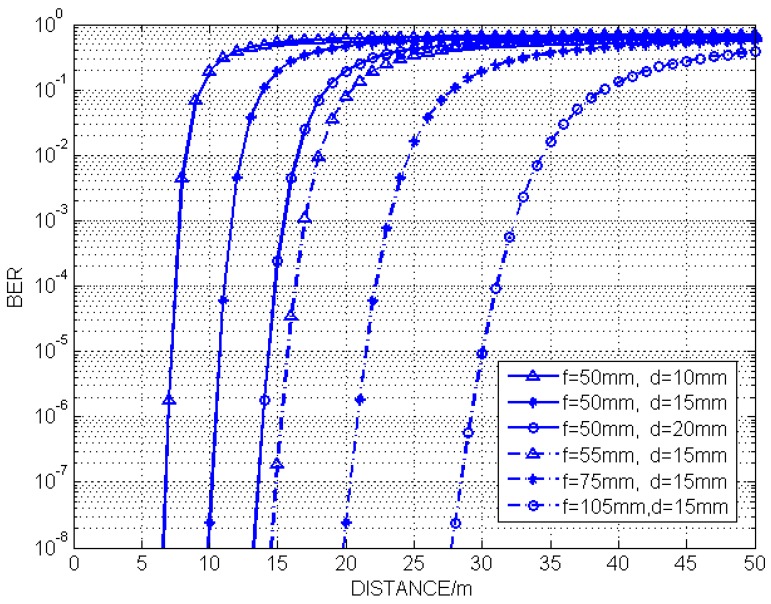
Bit error ratio performance of different communication distances.

In the simulation, the LED array size is 6×6. Figure 10 shows the simulation results of the system BER with different communication distances. The curve shows the same trend that the BER increases sharply when the system communication distance increases to a critical distance. Another trend is that the critical distance shifts to the right when the LED spacing distance *d* or the focal length *f* increases. The larger *d* the LED array has or the larger *f* the lenses have, the longer communication distance the system achieves. From Equations (6) and (10), the increase of the communication distance leads to the decrease of $r_{ij}^{\prime }$, which will significantly increase the system’s SISI noise. It means that the critical communication distance of the MIMO-IS-VLC system is mainly affected by the SISI noise intensity. The specific process of the whole experiment is described below.

It is known from Section 2 that the system stray light produces the SISI noise and the background noise component in the form of additive noise. The additive noise results in the degrading of the system BER performance. Generally, the background noise component can be calculated so that the fixed detection threshold is $T_{f}=\frac{GP_{LED}+\xi K}{2}$.

**Figure 11:**
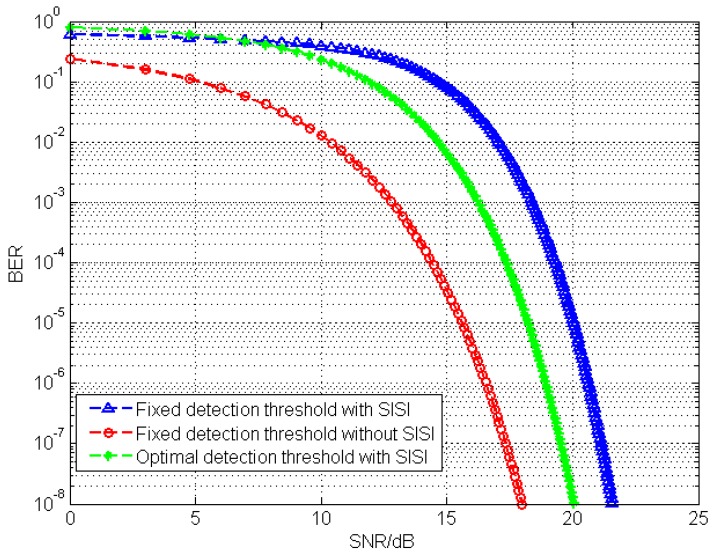
The impact of SISI and detection threshold on BER performance.

The ideal system BER performance with a fixed detection threshold is demonstrated in Figure 11. It is shown that, in a certain communication distance, the SISI noise reduces the BER performance remarkably. In order to define the optimal detection threshold, the distribution coefficient σ of the stray light PSF can be estimated by a fitting analysis tool. According to Equation (2) the SISI noise intensity can be estimated with the spacing distance d on the LED array, the lens’ focal length f, the certain communication distance u and the distribution coefficient σ. Hence, the optimal detection threshold can be calculated. In Figure 11, the simulation result indicates that, compared with the fixed detection threshold condition, the BER curve with an optimal detection threshold is closer to the ideal BER performance curve. In other words, by means of estimating the SISI noise intensity and adaptively adjusting the detection threshold, the system BER performance significantly improves.

**Figure 12:**
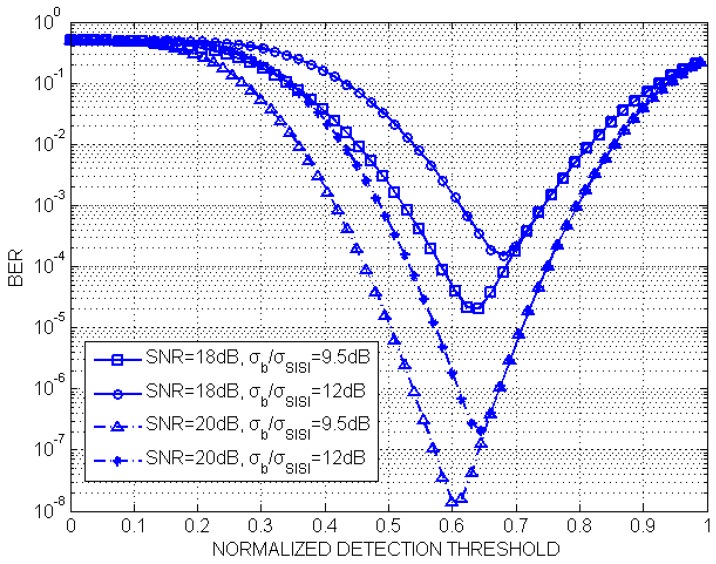
The BER curves of different focal lengths of the lenses.

Figure 12 shows that there exists an optimal normalized detection threshold that makes the system achieve the best BER performance. In the MIMO-IS-VLC system, the background noise mainly comes from the ambient light while the SISI noise is related to the received LED light power $P_{LED}^{\prime }$, the spacing distance *d* on the LED array, and the lens’ focal length. As a result, the background noise component and the SISI noise component are independent. The simulation result shows that the $\sigma_{b}\setminus \sigma_{SISI}$ influences the detection threshold value. If the $\sigma_{b}\setminus \sigma_{SISI}$ increases from 9.5 to 12 dB, the normalized detection threshold value increases approximately by 0.05 when the system SNR is 20 dB. In addition, it is indicated that, along with the decrease of the system SNR, the optimal normalized detection threshold increases.

## 4. Conclusions

In this paper, MIMO image sensors based on visible light communication are designed and researched from the perspective of optical communication systems. The formation mechanism of the stray light in the imaging system, the influence of the spatial inter-symbol interference, and RGB influence of RGB image sensors are analyzed. The stray light, caused by the incident light’s diffraction, refraction, and reflection produces the spatial inter-symbol interference. The SISI noise intensity is related to the received LED light power $P_{LED}^{\prime }$, the spacing distance *d* on the LED array, and the lens’ focal length *f*. Based on the imaging laws, the SISI noise intensity is directly correlated to the average expectation value of the spacing distance $r_{ij}^{\prime }$, which is the distance between two adjacent LEDs in the imaging plane. Consequently, the SISI noise intensity can be estimated by the given *d*, *f* and the certain communication distance *u*. The simulation results and analysis are given.

Furthermore, an adaptive threshold detection method is introduced and the performance is analyzed. The SISI noise influences the system BER performance in the form of additive noise. By means of estimating the SISI noise power, the optimal detection threshold can be obtained and adaptively adjusted so that the system BER performance enhances significantly. The simulation results and analysis indicate that $\sigma_{b}\setminus \sigma_{SISI}$, the ratio of the background noise intensity, and the SISI noise intensity determine the normalized optimal detection threshold value.

In summary, the modeling and analysis of SISI are significant to improve the MIMO-IS-VLC system performance. In the future work, the optical power to pixels’ intensity conversion will be taken into consideration so that the optimal image-binary threshold can be applied in the practical application to improve the system performance.

## Figures and Tables

**Table 1:** Common video format.

Video Output Specification	Resolution
Sub-quarter common intermediate format (sub-QCIF)	128 × 96
Quarter common intermediate format (QCIF)	176 × 144
Common intermediate format (CIF)	352 × 288
4 common intermediate format (4CIF)	704 ×76
16 common intermediate format (16CIF)	1408 × 1152
video graphics array (VGA)	640 × 480
Super video graphics array (SVGA)	800 × 600
Extended Graphics Array (XGA)	1024 × 768
Wide video graphics array (WVGA)	800 × 480
Super extended graphics array (SXGA)	1280 × 1024

**Table 2:** Experimental device of related parameters.

Pixel Size	2.2 μm × 2.2 μm
Image Area	3590 μm × 2684 μm
Maximum Image Transfer Rate of SXGA	15 fps
Maximum Image Transfer Rate of SVGA	30 fps
Maximum Image Transfer Rate of CIF	60 fps
Focal Length	20 mm
LED Array Distance	1.8 cm
